# Complex Disseminated Tuberculosis with Oral and Gastrointestinal Involvement: Histopathologic and Clinical Insights

**DOI:** 10.3390/diagnostics16050727

**Published:** 2026-02-28

**Authors:** Nicoleta Zurbău, Imola Miklos, Laura Ioana Bondar, Denis Bogdan Butari, Florin Mihai Șandor, Maria Daniela Moț, Ana-Liana Bouroș Tătaru, Nilima Rajpal Kundnani, Casiana Boru, Paula Irina Barata

**Affiliations:** 1 Faculty of Medicine, “Vasile Goldis” Western University of Arad, 94–96 Revolutiei Avenue, 310025 Arad, Romania; zurbau.nicoleta@uvvg.ro; 2 Department of Physiology, Faculty of Medicine, “Vasile Goldis” Western University of Arad, Blvd. Revolutiei, No. 96, 310045 Arad, Romania; miklos.imola@uvvg.ro (I.M.); barata.paula@uvvg.ro (P.I.B.); 3 Doctoral School of Biomedical Sciences, University of Oradea, University Street, No. 1, 410087 Oradea, Romania; bondar.lauraioana@student.uoradea.ro; 4 Department of Biology and Life Sciences, Faculty of Medicine, “Vasile Goldis” Western University of Arad, Blvd. Revolutiei, No. 96, 310025 Arad, Romania; butari.denis-bogdan@uvvg.ro (D.B.B.); sandor.florin@uvvg.ro (F.M.Ș.); 5 Department of General Medicine, “Vasile Goldis” Western University of Arad, Blvd. Revolutiei, No. 96, 310025 Arad, Romania; 6 Department of Cardiology-Internal Medicine and Ambulatory Care, Prevention and Cardiovascular Recovery, “Victor Babes” University of Medicine and Pharmacy, 300041 Timisoara, Romania; knilima@umft.ro; 7 Department of Medicine, “Vasile Goldis” University of Medicine and Pharmacy, 310414 Arad, Romania; boru.casiana@uvvg.ro

**Keywords:** tuberculosis, miliary tuberculosis, oral tuberculosis, granulomatous inflammation, differential diagnosis

## Abstract

**Background and Clinical Significance:** Extrapulmonary tuberculosis (TB) remains a diagnostic challenge, particularly when affecting rare sites such as the oral cavity and digestive tract. We report the case of a 55-year-old woman with disseminated (miliary) tuberculosis presenting with atypical oral lesions initially suspected to represent a malignant tumor. **Case Presentation:** The patient had a history of recurrent depressive disorder, cognitive impairment, sleep disturbances, and nicotine/alcohol dependence. She presented with painful ulcerations of the oral cavity, dysphagia, odynophagia, and glossodynia. Otolaryngologic examination revealed reduced tongue mobility and an ulceroinfiltrative lesion involving the floor of the mouth and the lower alveolar ridge. Fibroscopic evaluation confirmed infiltrative ulcerative lesions, and biopsy samples were obtained. Histopathologic examination revealed a chronic necrotizing granulomatous inflammation with multinucleated giant cells, consistent with a mycobacterial infection. Further investigations confirmed disseminated (miliary) tuberculosis with oral and digestive involvement. Antituberculous therapy was initiated; however, despite temporary stabilization, the patient’s condition progressively worsened and the outcome was fatal. **Conclusions:** Oral and digestive tuberculosis, although rare, should be considered in the differential diagnosis of ulceroinfiltrative lesions of the oral cavity, particularly in patients with systemic symptoms or risk factors for TB. Early histopathologic confirmation and initiation of specific therapy are essential for favorable outcomes and prevention of misdiagnosis as malignant disease.

## 1. Introduction

Tuberculosis (TB) remains one of the leading infectious causes of death globally, with an estimated 10.6 million new cases and 1.3 million deaths reported in 2023 according to the World Health Organization (WHO) [1]. Despite sustained public health efforts, the global TB burden has been exacerbated by socioeconomic instability, underdiagnosis, and the spread of resistant strains. In Europe, TB incidence has continued to decline overall, but the proportion of extrapulmonary tuberculosis (EPTB) has increased, particularly among immunocompromised individuals and those with chronic comorbidities [2]. In Romania, one of the highest TB-burden countries in the European Union, EPTB represents approximately 12–15% of reported cases, highlighting the ongoing diagnostic and public health challenges. [WHO Global TB Report 2024; ECDC Surveillance Report 2025] [3].

Extrapulmonary TB can affect virtually any organ, most frequently the lymph nodes, pleura, bones, meninges, and genitourinary tract. The miliary form, resulting from hematogenous dissemination of *Mycobacterium tuberculosis*, involves multiple organs simultaneously and carries a significant risk of fatal outcomes if untreated [4]. Miliary tuberculosis is often underrecognized due to its polymorphic presentation and nonspecific systemic manifestations, such as prolonged fever, malaise, and weight loss, which can mimic malignancies or autoimmune diseases [5].

Diagnosis of EPTB remains difficult even in high-resource settings, as the disease may present with low bacterial load and atypical imaging findings. Modern diagnostic tools—such as GeneXpert MTB/RIF Ultra, line probe assays, and interferon-gamma release assays (IGRAs)—have improved sensitivity, but tissue biopsy with histopathological and microbiological confirmation remains the gold standard for diagnosis. [WHO Consolidated Guidelines on TB Diagnosis 2023] [6].

Among EPTB manifestations, oral tuberculosis is exceedingly rare, accounting for less than 1% of all TB cases. It may occur as a primary infection or, more commonly, as a secondary manifestation of pulmonary or disseminated disease [7]. Oral TB lesions often present as chronic ulcerative or infiltrative masses involving the tongue, gingiva, or floor of the mouth. Clinically and macroscopically, these lesions closely resemble squamous cell carcinoma or chronic traumatic ulcers, frequently leading to initial misdiagnosis. Delay in recognition is common, as TB is rarely considered in the differential diagnosis of oral mucosal lesions, especially in immunocompetent patients [8].

Given the resurgence of extrapulmonary and disseminated forms of tuberculosis, awareness of such atypical presentations is essential. A multidisciplinary approach involving otolaryngologists, infectious disease specialists, and pathologists is critical for early recognition and initiation of antituberculous therapy, which can dramatically improve outcomes even in cases mimicking malignancy [9].

In Romania, tuberculosis remains a major public health concern despite a sustained national control program. The country continues to report the highest TB incidence in the European Union, with approximately 44 cases per 100,000 population in 2024, well above the EU average. According to the Romanian National Institute of Public Health and the WHO, an estimated 9500–10,000 new TB cases are diagnosed annually. Although TB mortality has decreased by nearly 70% over the past two decades, transmission persists, particularly among socioeconomically vulnerable groups. Pediatric incidence remains elevated, reaching about 10 cases per 100,000 children in 2024, indicating ongoing community spread. Moreover, multidrug-resistant tuberculosis (MDR-TB) continues to pose a significant challenge, emphasizing the need for early detection and comprehensive management strategies. These trends highlight that, even in countries with established TB programs, diagnostic delays and underrecognition of extrapulmonary and atypical forms—such as oral and digestive TB—remain major barriers to effective control [10,11,12].

## 2. Materials and Methods

A 55-year-old woman from Arad, Romania, employed in a factory with rotating shifts, was evaluated for chronic ulcerations of the oral cavity, dysphagia, odynophagia, and glossodynia persisting for approximately two years. Her medical history included recurrent depressive disorder, cognitive impairment, sleep disturbances, and nicotine and alcohol dependence. The patient had received Bacille Calmette–Guérin (BCG) vaccination in early childhood according to the Romanian national immunization schedule. Over this period, she had received multiple courses of antibiotics prescribed by dental practitioners, which provided only transient symptom relief. The patient’s symptoms began approximately two years prior to diagnosis, with recurrent oral ulcerations and progressive dysphagia. The clinical timeline, from symptom onset to surgery and death, is summarized chronologically below. Multiple evaluations by dental and primary care practitioners resulted in repeated antibiotic courses without sustained improvement. Following referral to the otorhinolaryngology department, a detailed diagnostic work-up—including fibroscopy, biopsy, chest and abdominal CT, and microbiological testing—confirmed disseminated tuberculosis. The clinical sequence, investigations, and therapeutic interventions are now described chronologically for clarity.

Before tuberculosis was suspected, the patient received several empiric antibiotic regimens prescribed by dental practitioners, including amoxicillin–clavulanate, clindamycin, and later levofloxacin, over multiple short courses. These treatments produced only transient symptom relief, contributing to diagnostic delay. Due to persistent oral pain and progressive ulceration, the patient was referred to the otorhinolaryngology (ENT) department, where clinical examination revealed reduced tongue mobility and an ulceroinfiltrative lesion involving the floor of the mouth and the lower alveolar ridge. Given the infiltrative appearance, an oral cavity malignancy was initially suspected. Fibroscopic assessment confirmed extensive ulcerative and indurated lesions, and biopsy specimens were obtained.

Histopathologic analysis demonstrated chronic necrotizing granulomatous inflammation with multinucleated giant cells consistent with *Mycobacterium tuberculosis* infection. Subsequent chest computed tomography (CT) revealed a miliary pattern with diffuse micronodular opacities throughout both lungs, establishing the diagnosis of miliary tuberculosis. Sputum cultures were positive for *Mycobacterium tuberculosis* (BK-positive).

Antituberculous therapy was initiated with rifampicin, pyrazinamide, ethambutol, and levofloxacin. Isoniazid was omitted due to the patient’s concurrent psychiatric treatment (sertraline, mirtazapine, alprazolam, trazodone, pramistar, and piracetam) and the potential for adverse neuropsychiatric interactions. Serial laboratory monitoring revealed marked thrombocytosis, leukocytosis, and anemia. The patient developed severe abdominal pain and recurrent vomiting during therapy, initially attributed to drug intolerance. However, abdominal CT demonstrated intestinal obstruction involving the terminal ileum and proximal cecum. Emergency surgery was performed, and histopathologic examination of the resected segment confirmed intestinal tuberculosis.

The patient continued antituberculous therapy postoperatively with gradual clinical stabilization.

Informed consent was obtained from the patient for publication of this case report. All procedures and documentation adhered to institutional ethical guidelines and the Declaration of Helsinki.

## 3. Results

**Figure 1:**
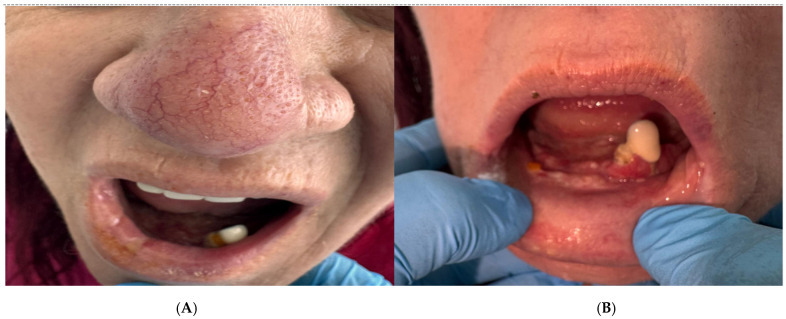
Clinical presentation of an oral lesion. (**A**) External view showing swelling and ulceration at the level of the lower lip. (**B**) Intraoral examination revealing an ulcerated, exophytic lesion of the oral mucosa.

The patient presented with chronic oral cavity ulcerations, dysphagia, odynophagia, and glossodynia persisting for approximately two years. Otorhinolaryngologic examination revealed reduced tongue mobility and an ulceroinfiltrative lesion involving the floor of the mouth and the lower alveolar ridge. Given the infiltrative appearance, an oral malignancy was initially suspected. Fibroscopic assessment confirmed extensive ulcerative and indurated lesions (Figure 1).

**Figure 2:**
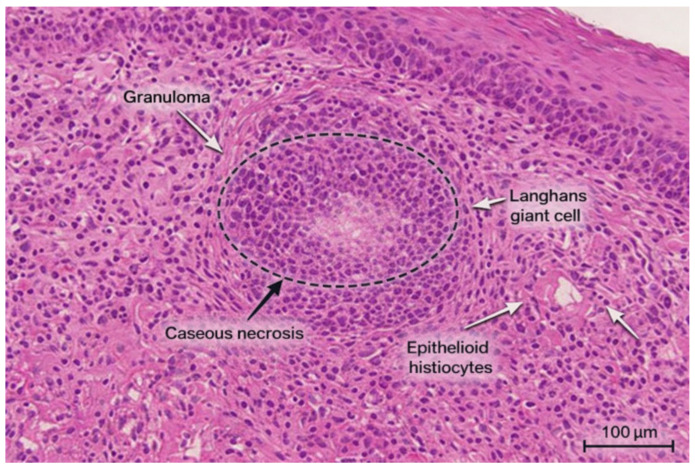
Histopathological examination of the oral biopsy showing chronic necrotizing granulomatous inflammation with central caseous necrosis (black arrow), surrounded by epithelioid histiocytes and Langhans-type multinucleated giant cells (white arrows). Hematoxylin–Eosin stain, original magnification ×200. Scale bar = 100 μm.

Histopathologic examination of oral biopsy specimens demonstrated a chronic necrotizing granulomatous inflammation with multinucleated giant cells. The squamous non-keratinized epithelium was hyperplastic with spongiosis and abundant neutrophilic exocytosis. The corium was expanded by chronic granulomatous inflammation, with focal caseous necrosis surrounded by epithelioid histiocytes and numerous Langhans-type multinucleated giant cells, consistent with mycobacterial infection (Figure 2).

Although hematoxylin–eosin staining revealed necrotizing granulomatous inflammation with Langhans-type giant cells, Ziehl–Neelsen staining additionally demonstrated acid-fast bacilli within the oral lesion, confirming a mycobacterial etiology. This histopathologic finding, in conjunction with positive sputum culture and GeneXpert MTB/RIF confirmation of *Mycobacterium tuberculosis*, established the diagnosis of disseminated tuberculosis rather than other granulomatous infections. While culture from the oral lesion was not feasible due to limited viable tissue, Ziehl–Neelsen staining and PCR testing on biopsy samples detected acid-fast bacilli and *M. tuberculosis* DNA, respectively, confirming local tuberculous involvement.

The differential diagnosis of the patient’s oral lesions included malignancy, fungal infection, syphilis, and Crohn’s disease. Malignancy was excluded based on histopathology showing granulomatous inflammation without atypical cells. Fungal infection was ruled out by negative PAS and GMS staining. Syphilis was excluded through negative serology (VDRL and TPHA). Crohn’s disease was considered unlikely due to the absence of typical intestinal lesions and lack of granulomatous inflammation patterns specific to Crohn’s disease on biopsy. This systematic exclusion supports the final diagnosis of oral involvement by disseminated tuberculosis. Molecular testing confirmed Mycobacterium tuberculosis infection via GeneXpert PCR on oral and intestinal biopsy samples. Acid-fast bacilli (AFB) staining of tissue sections was positive, supporting active infection. Drug susceptibility testing indicated sensitivity to first-line antituberculous drugs, guiding the subsequent tailored therapeutic regimen.

**Figure 3:**
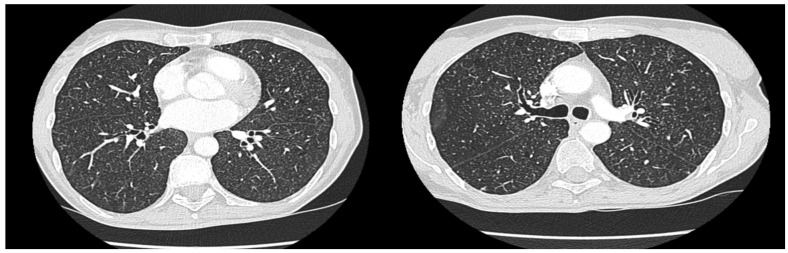
Chest CT showing micronodular lesions consistent with miliary TB.

Chest computed tomography (CT) revealed (Figure 3):

- Micronodular pulmonary lesions compatible with miliary tuberculosis.
- Apical bilateral sequelae of previous TB.
- Mediastinal millimetric lymphadenopathy.
- No pleural or pericardial effusion.
- Tracheobronchial axes free.

Sputum examination was positive for *Mycobacterium tuberculosis* (BK-positive). Serologic tests for VDRL/TPHA and HIV (ELISA + Western Blot) were negative.

Laboratory findings at presentation included marked anemia, leukocytosis with neutrophilia, and thrombocytosis, consistent with chronic inflammation and ongoing infection, along with significant hepatocellular injury likely exacerbated by TB therapy. Fecal occult blood was positive, correlating with gastrointestinal involvement (Table 1).

Given the marked elevation of liver transaminases (AST 1075 U/L, ALT 1120 U/L), antituberculous therapy was temporarily discontinued and supportive hepatoprotective treatment was initiated, including intravenous hydration, vitamin supplementation, and close biochemical monitoring. After partial normalization of liver function, therapy was cautiously reintroduced with a modified regimen excluding isoniazid and adjusting doses of rifampicin and pyrazinamide. Despite these measures, hepatocellular injury persisted, reflecting the cumulative burden of disseminated infection, polypharmacy, and impaired hepatic reserve. It is important to distinguish that while drug-related hepatotoxicity and gastrointestinal intolerance affected medication tolerance, the intestinal obstruction was a direct consequence of gastrointestinal tuberculosis progression, as confirmed by imaging and histopathology.

The relationship between antituberculous therapy and adverse events was notable: rifampicin and pyrazinamide likely contributed to hepatotoxicity and gastrointestinal discomfort, while levofloxacin exacerbated digestive symptoms. Anemia, thrombocytosis, and leukocytosis were attributed to chronic inflammation, gastrointestinal blood loss, and active infection.

**Figure 4:**
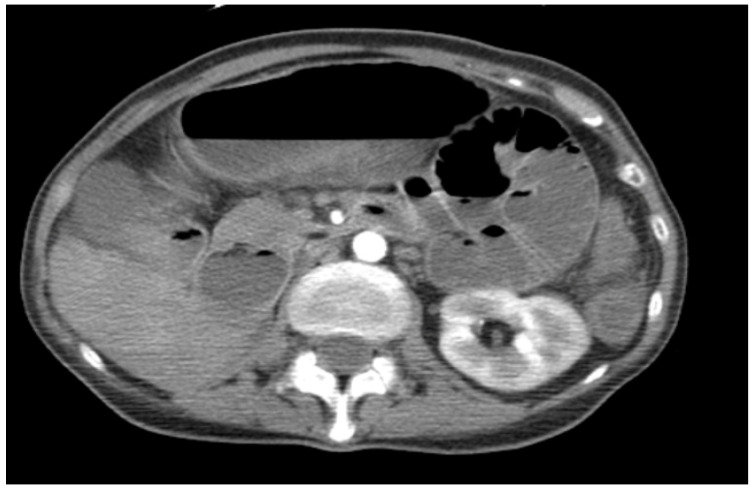
Abdominal CT showing ileocecal wall thickening with small bowel distension and stasis, perienteric fat stranding, and hepatomegaly; other abdominal organs appear normal.

Abdominal and pelvic CT demonstrated (Figure 4):

- Circumferential wall thickening (14 mm) of the terminal ileum extending into the proximal cecum.
- Distended small bowel loops with intraluminal stasis and air-fluid levels.
- Perienteric fat stranding without pneumoperitoneum.
- Gastric and esophageal stasis.
- Hepatomegaly with two small cysts (segments V and VII) and diffuse steatosis.
- Normal spleen, pancreas, and adrenal glands.
- Single functioning left kidney.

**Figure 5:**
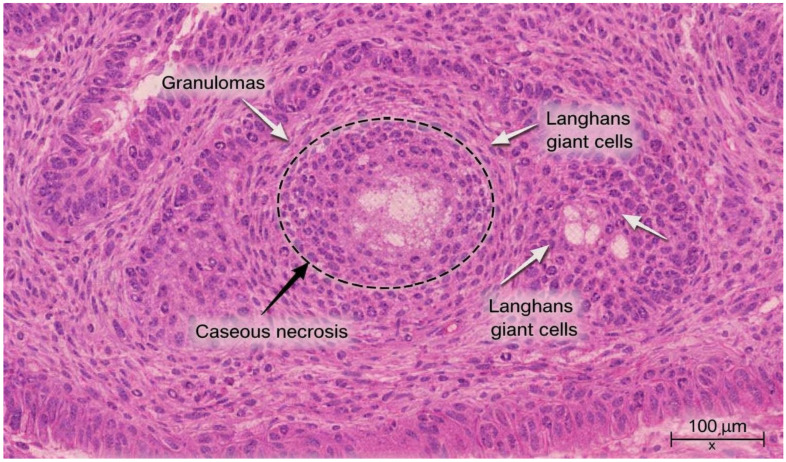
Histopathological examination of the ileocecal biopsy demonstrating confluent epithelioid granulomas with central caseous necrosis (black arrow) and Langhans-type multinucleated giant cells (white arrows), consistent with tuberculosis. Hematoxylin–Eosin stain, original magnification ×200; scale bar = 100 μm.

The patient developed severe nausea, vomiting, and worsening general condition during antituberculous therapy (rifampicin, pyrazinamide, ethambutol, and levofloxacin). Initial attribution to drug intolerance led to temporary treatment discontinuation. Emergency surgical intervention was performed due to intestinal obstruction. Histopathologic examination of ileocecal biopsy confirmed tuberculous granulomatous inflammation with multiple confluent epithelioid granulomas, central caseous necrosis, and Langhans-type multinucleated giant cells (Figure 5).

Overall, the patient’s condition was severe, reflecting the complex interplay of miliary TB, extrapulmonary involvement (oral and intestinal), comorbidities, and drug-related toxicity. Following surgical intervention and continuation of adjusted TB therapy, gradual clinical improvement was observed.

The patient developed intestinal obstruction, necessitating emergency surgical intervention. Antituberculous therapy was cautiously reintroduced with a modified regimen excluding isoniazid and adjusting doses of rifampicin and pyrazinamide. Despite these measures, her hepatic enzymes remained elevated and clinical condition was unstable. Despite emergency surgical intervention and continuation of adjusted antituberculous therapy, the patient’s clinical condition deteriorated rapidly, and she unfortunately succumbed on the first postoperative day. This outcome highlights the severity of disseminated miliary tuberculosis with multiple extrapulmonary localizations and the challenges associated with managing complex cases in patients with significant comorbidities.

## 4. Discussion

This case underscores the diagnostic complexity associated with extrapulmonary tuberculosis (EPTB), particularly when presenting with rare oral and gastrointestinal localizations. As in other reports, the nonspecific clinical presentation contributed to delayed recognition. Nonspecific symptoms such as chronic ulcerations, dysphagia, and abdominal pain frequently mimic more common conditions, leading to initial misdiagnosis and progression to serious complications such as intestinal obstruction that necessitate urgent surgical intervention. Similar diagnostic challenges have been described in the literature, including cases where miliary TB with oral and intestinal involvement presented initially as unexplained lesions or masses requiring biopsy for clarification [13,14].

What makes this case particularly distinctive is the simultaneous involvement of both the oral cavity and the gastrointestinal tract two exceedingly rare extrapulmonary sites combined with an unusually long two-year diagnostic delay and a fatal outcome despite appropriate therapy.

Although oral granulomatous inflammation may occur in mycoses or nocardiosis, ancillary investigations—including PAS and Grocott stains (negative for fungal elements) and absence of Nocardia on culture—excluded these possibilities. The detection of M. tuberculosis DNA in the oral specimen confirmed a tuberculous etiology consistent with disseminated disease [10,12].

Tuberculosis involving the oral cavity and digestive tract is rare and often overlooked, particularly in regions where TB prevalence is low or where clinicians have low index of suspicion. Oral TB lesions can closely resemble malignancies or other chronic ulcerative conditions, leading to extensive differential diagnostic work-ups before TB is considered. In this case, the initial clinical and fibroscopic findings raised suspicion for a primary oral cavity neoplasm, which is consistent with prior case reports describing similar presentations [15].

Definitive diagnosis in extrapulmonary TB cases often relies on histopathological examination. The detection of necrotizing epithelioid granulomas with Langhans-type multinucleated giant cells is characteristic and serves as a cornerstone for diagnosing TB when microbiological results are pending or inconclusive. Histopathology played a crucial role in clarifying the diagnosis in this case, as in many previously published reports of oral TB and extrapulmonary involvement [16,17].

The presence of pulmonary micronodules consistent with miliary tuberculosis and widespread extrapulmonary involvement highlights the severe systemic nature of the disease [18]. While miliary TB is typically paucibacillary and often difficult to confirm microbiologically, positive sputum results in this patient—both on initial smear and in prolonged culture—are notable and have been documented in selected cases of disseminated TB with active pulmonary lesions. This emphasizes that, although unusual, positive bacteriological findings can occur even in disseminated forms, aiding diagnostic confirmation [19].

Gastrointestinal TB, particularly ileo–cecal involvement, is known to present with nonspecific symptoms such as abdominal pain and obstruction, often mimicking Crohn’s disease or malignancy [20]. Published reports describe similar diagnostic pitfalls and indicate that ileocecal TB remains an important differential diagnosis in regions with higher TB prevalence or in immunocompromised populations. The ileocecal obstruction in this case represents a severe manifestation requiring emergent surgical management, aligning with previous literature emphasizing the need for timely recognition to prevent catastrophic outcomes [21].

The management of TB in patients with significant comorbidities and polypharmacy is particularly challenging. The intolerance and adverse gastrointestinal effects observed in this patient complicated antituberculous therapy [22]. The exclusion of isoniazid from the regimen, due to concerns regarding potential drug–drug interactions with psychiatric medications and risk of neuropsychiatric toxicity, may have reduced regimen potency. Although including isoniazid in standard therapy is fundamental, balancing the risks of interactions and exacerbation of psychiatric conditions is complex, especially as treatment interruptions themselves are risk factors for poor outcomes [23]. Severe drug-induced hepatotoxicity represented a major therapeutic challenge in this case. The degree of hepatic enzyme elevation (>1000 U/L) is consistent with grade 4 hepatotoxicity according to WHO criteria and required temporary discontinuation of therapy and supportive management. However, the combination of ongoing disseminated infection and compromised hepatic metabolism likely contributed to irreversible hepatic failure and overall clinical deterioration [22,23].

Despite definitive diagnosis, targeted therapy, and surgical intervention, the patient’s clinical course was unfavorable, resulting in death in the early postoperative period. This outcome aligns with reports showing that disseminated TB with multiple organ involvement carries a high mortality risk, particularly when diagnosis and treatment initiation are delayed. A case of miliary TB with gastrointestinal bleeding also documented a fatal outcome despite intervention, underscoring the severity of such presentations even with current diagnostic and therapeutic strategies [24].

In conclusion, this case emphasizes the importance of maintaining a high index of suspicion for TB in patients with atypical manifestations, the central role of histopathologic and microbiological confirmation, and the significant challenges in managing complex disseminated disease, particularly in the setting of comorbid conditions and polypharmacy. Early recognition and comprehensive multidisciplinary management are essential to improve outcomes in similar future cases [25].

This fatal outcome, despite timely surgical management, reflects the severe systemic burden and rapid progression characteristic of disseminated miliary tuberculosis.

### Clinical Take-Home Messages

1. High suspicion is crucial: Oral and gastrointestinal tuberculosis are rare and often mimic malignancy or other chronic inflammatory conditions; delayed recognition can lead to severe complications.
2. Histopathology remains essential: Biopsy demonstrating necrotizing granulomatous inflammation with Langhans-type giant cells is often the key to definitive diagnosis in atypical TB presentations.
3. Miliary TB can be microbiologically positive: Even though miliary TB is typically paucibacillary, positive sputum smear and prolonged culture can occur and aid in confirming the diagnosis.
4. Management complexity: Comorbidities, polypharmacy, and drug intolerance may necessitate modified treatment regimens, which can impact therapy efficacy and prognosis.
5. Rapid deterioration is possible: Disseminated TB with multiple extrapulmonary localizations carries a high risk of severe complications and mortality, highlighting the importance of timely diagnosis and multidisciplinary management.

## 5. Conclusions

Intestinal and oral tuberculosis present significant diagnostic challenges due to their nonspecific clinical manifestations, which may mimic inflammatory bowel disease or malignancy. Early recognition requires a high index of suspicion and careful correlation of clinical findings, imaging studies, and histopathology [26].

Histopathological confirmation remains essential, with necrotizing epithelioid granulomas and Langhans-type giant cells providing pathognomonic evidence. The use of molecular diagnostic tests on oral or gastrointestinal specimens may further enhance bacteriological sensitivity and facilitate timely diagnosis [27].

Severe gastrointestinal complications, such as ileocecal obstruction, may necessitate urgent surgical intervention. Delayed diagnosis increases the risk of morbidity and the need for invasive procedures [28].

Although standard antituberculous therapy is generally effective, drug intolerance and interruptions—due to hepatic or gastrointestinal adverse effects—can negatively affect prognosis and promote disease dissemination. This underscores the importance of strict monitoring and a multidisciplinary approach, involving infectious disease specialists, gastroenterologists, surgeons, and pathologists [29].

The presented case illustrates several key points documented in the literature: initial presentation as an oral lesion mimicking a tumor, histopathologic confirmation of tuberculosis, association with miliary pulmonary dissemination, and severe intestinal complications. The unfavorable outcome, related to treatment interruptions and drug intolerance, emphasizes the critical importance of treatment adherence, early diagnosis, and comprehensive management in complex disseminated TB cases [30,31].

Practical implications for clinicians include:

- Maintaining a high index of suspicion for TB in atypical oral lesions or ileocecal masses;
- Obtaining multiple tissue or microbiological samples for accurate diagnosis;
- Close monitoring of drug-related adverse effects;
- Early involvement of a multidisciplinary team to prevent and manage complications.

## Figures and Tables

**Table 1:** Key laboratory parameters over 27–29 August 2025.

Parameter	27 August 2025	28 August 2025	29 August 2025	Reference Range
Hemoglobin (HGB)	9.90 g/dL	9.50 g/dL	9.10 g/dL	12–16 g/dL
Hematocrit (HCT)	27.9%	26.8%	25.7%	36–46%
Red blood cells (RBC)	3.54 × 10^6^/µL	3.42 × 10^6^/µL	3.30 × 10^6^/µL	4.2–5.4 × 10^6^/µL
White blood cells (WBC)	34.55 × 10^3^/µL	36.20 × 10^3^/µL	37.80 × 10^3^/µL	4–10 × 10^3^/µL
Neutrophils (abs.)	29.38 × 10^3^/µL	30.80 × 10^3^/µL	32.10 × 10^3^/µL	2–7 × 10^3^/µL
Neutrophils (%)	85.1%	85.2%	85.0%	40–70%
Lymphocytes (abs.)	0.89 × 10^3^/µL	0.82 × 10^3^/µL	0.76 × 10^3^/µL	1–4 × 10^3^/µL
Lymphocytes (%)	2.6%	2.3%	2.0%	20–45%
Monocytes (%)	8.3%	8.5%	8.7%	2–8%
Platelets (PLT)	698 × 10^3^/µL	710 × 10^3^/µL	725 × 10^3^/µL	150–450 × 10^3^/µL
RDW-SD	58.5 fL	59.0 fL	59.5 fL	37–54 fL
RDW-CV	18.5%	18.8%	19.0%	11–15%
MCH	28.0 pg	27.8 pg	27.5 pg	27–32 pg
MCHC	29.7 g/dL	29.3 g/dL	29.0 g/dL	32–36 g/dL
TGO (AST)	978 U/L	1020 U/L	1075 U/L	10–40 U/L
TGP (ALT)	1024 U/L	1070 U/L	1120 U/L	10–40 U/L
Uric acid	18 mg/dL	18.5 mg/dL	19 mg/dL	3.5–7.2 mg/dL
Fecal occult blood	Positive	-	-	Negative

## Data Availability

The data presented in this study are available on request from the corresponding author.

## Abbreviations

ALT/TGP	Alanine aminotransferase
AST/TGO	Aspartate aminotransferase
BK	Bacillus Koch (Mycobacterium tuberculosis)
CT	Computed Tomography
EPTB	Extrapulmonary Tuberculosis
HCT	Hematocrit
HGB	Hemoglobin
IGRA	Interferon-Gamma Release Assay
MDR-TB	Multidrug-Resistant Tuberculosis
PLT	Platelets
RBC	Red Blood Cells
RDW-CV/RDW-SD	Red Cell Distribution Width–Coefficient of Variation/Standard Deviation
TB	Tuberculosis
VDRL/TPHA	Venereal Disease Research Laboratory/Treponema pallidum Hemagglutination Assay
WBC	White Blood Cells
